# A Response to Blanc and Colleagues’ Viewpoint on Gestalt Language Processing and the Natural Language Acquisition Protocol: Concerns and Common Ground

**DOI:** 10.1044/2025_persp-25-00055

**Published:** 2025-12-01

**Authors:** Emily Lorang, Janine Mathée-Scott, Jennifer Johnson, Courtney E. Venker

**Affiliations:** a Department of Communicative Sciences and Disorders, Michigan State University, East Lansing; b Department of Speech Pathology and Audiology, Marquette University, Milwaukee, WI

## Abstract

Gestalt Language Processing (GLP) and the Natural Language Acquisition (NLA) protocol have recently grown in popularity in the field of speech-language pathology. However, significant controversy surrounds the ideas proposed and clinical strategies recommended within GLP/NLA. In this commentary, we respond to the *Perspectives of the ASHA Special Interest Groups* viewpoint by Blanc et al. (2023) detailing the hypothesized alternative path of language development, proposed stages, and recommended supports associated with GLP/NLA. We identify several strategies recommended by the NLA protocol that are also part of general developmental clinical practices. We go on to describe five concerns associated with GLP/NLA: It is contradicted by existing research, has a fundamental conceptual flaw, presumes incompetence, recommends strategies that may be harmful, and lacks supporting research evidence. We close by emphasizing the importance of engaging in respectful and professional conversations about GLP/NLA and using precise language to describe its individual components.

On December 7, 2023, the American Speech-Language-Hearing Association (ASHA) Special Interest Group 1 published a viewpoint titled “Using the Natural Language Acquisition Protocol to Support Gestalt Language Development” (Blanc et al., 2023). This article was the most read article published in any ASHA journal in 2023—a designation that illustrates the current popularity of Gestalt Language Processing (GLP) and the Natural Language Acquisition (NLA) protocol within the field of speech-language pathology (ASHA, n.d.). GLP/NLA is a collection of ideas and clinical strategies that are commonly discussed in relation to autistic children’s production of delayed echolalia—word-for-word repetitions of previously heard speech (Blanc et al., 2023). GLP is built on the assumption that delayed echolalia indicates a GLP style, which Blanc et al. (2023) describe as “… a style of language acquisition that begins with larger chunks of language, as opposed to the traditional style of language acquisition (known as ‘analytic language processing’ [ALP]) that begins with single words” (p. 1280). Blanc et al. assert that “… autistic language development often follows the gestalt style of language acquisition” (p. 1279), and they refer to children believed to acquire language in a gestalt style as gestalt language processors. In the NLA protocol, Blanc et al. outline what they consider to be the six stages of gestalt language development and describe the intervention strategies they believe should be used for gestalt language processors at each stage.

In recent years, GLP and the NLA protocol have become increasingly popular among speech-language pathologists (SLPs) who work with autistic children (Evans, 2022, 2025; Hutchins et al., 2024). However, GLP and NLA have also begun to generate controversy related to the incorporation of concepts and clinical recommendations that lack theoretical and empirical support, and may have unintended negative consequences (Bryant et al., 2025; Evans, 2022, 2025; Hutchins et al., 2024; language_processing, n.d.; Venker & Lorang, 2024). It is important for clinicians to be aware of the concerns that have been raised, as this information may affect their views of GLP and NLA and the extent to which they use associated ideas and recommendations in their practice. Because GLP and NLA contain intimately linked and overlapping ideas, we often refer to GLP/NLA together.

In this commentary, we describe five concerns about GLP/NLA:

1. It is contradicted by existing research evidence showing that autistic children process spoken utterances incrementally (i.e., word-by-word), not as larger “chunks.”
2. It makes claims about children’s language processing “style” based on their spoken language production, which is a fundamental conceptual flaw.
3. It assumes autistic children do not understand individual words in their delayed echoes, which presumes incompetence and is inconsistent with neurodiversity.
4. It recommends unfounded and potentially harmful language modeling strategies (e.g., eliminating verbs from adult language input).
5. It is not supported by peer-reviewed, empirical research.

Despite these concerns, we recognize that GLP/NLA may have resonated with many SLPs because it is associated with numerous developmental strategies and general developmental clinical practices (see Table 1). For example, the NLA protocol encourages clinicians to follow the child’s lead, partner with families, embrace the communicative value of echolalia, and listen to autistic voices—strategies that prioritize authentic relationships with clients and their families and are consistent with neurodiversity (Blanc et al., 2023; Haydock et al., 2024). We firmly believe that these general developmental practices can (and should) continue to be used; none of the concerns we raise here pertain to these ideas. In particular, we emphasize that we unequivocally support the value of embracing echolalia as a form of autistic communication. However, these strategies and general developmental practices are not unique to the NLA protocol. They can be used independent of the NLA protocol and are included in many evidence-based interventions designed for autistic children (Binns et al., 2021; Bryant et al., 2025; Evans, 2022; Gaddy & Crow, 2023; Hutchins et al., 2024; Ingersoll & Dvortcsak, 2019; Izuno-Garcia et al., 2023; Lai et al., 2020; Paul et al., 2018; Rogers et al., 2012; Schreibman et al., 2015; Sone et al., 2021; Sussman, 2012; Venker & Lorang, 2024; Weitzman, 2013). Thus, the concepts associated with GLP/NLA cannot be adopted or discarded in an all-or-nothing fashion; they must each be evaluated independently.

We recognize that a lot of information is currently available, either supporting or opposing various aspects of GLP/NLA (Beals, 2024; Blanc et al., 2023; Brannick, 2025; Bryant et al., 2025; Evans, 2022, 2025; Haydock et al., 2024; Hutchins et al., 2024; Venker & Lorang, 2024). Our goal in writing this commentary is to provide a thorough analysis of the individual components and clinical recommendations associated with GLP/NLA. We hope that this information will support SLPs in making informed decisions about GLP/NLA, thereby maximizing our ability to provide evidence-based and neurodiversity-affirming practices for autistic people and their families. While we primarily respond to Blanc et al. (2023), we include additional sources at times to provide a more comprehensive picture of the information about GLP/NLA available to SLPs and families. The inclusion of online sources (e.g., websites, social media) is particularly relevant given that SLPs and parents often seek out clinical information online (Bryan et al., 2020; Diehm & Hall-Mills, 2023; Frey et al., 2022).

Generally speaking, new ideas about autism and language development are exciting because they have the potential to move the field forward—especially when they are informed by clinical experiences and observations. However, a set of ideas as complex as GLP/NLA cannot simply be proposed and accepted without evaluating its conceptual foundation and considering the extent to which it is supported or refuted by peer-reviewed, empirical research. If we do not fully evaluate new ideas in this way, we run the risk of adopting practices that are not in the best interest of the individuals we serve. Here, we describe five concerns about GLP/NLA.

## Concern 1: The Idea of GLP Is Contradicted by Existing Research on How Autistic Children Process Language

A key idea within GLP is that many autistic children process spoken utterances as “chunks” or as “an unanalyzed whole, that is, they are not aware that a language gestalt could be made up of individual words that are combined” (Blanc et al., 2023, p. 1280; see also Blanc, 2012; Peters, 1977).^1^ Language processing refers to the comprehension of speech as it unfolds (Huettig et al., 2011; Venker et al., 2013; Zhou et al., 2019). Although language processing has historically been difficult to measure in a clinical setting, eye-tracking methodologies provide the tools to measure moment-by-moment language processing (at the level of milliseconds) in a research context (Huettig et al., 2011; Su & Naigles, 2019; Venker & Kover, 2015; Zhou et al., 2019). Using this methodology, studies that collectively included over 300 children have shown that autistic children aged 3–17 years with a wide range of cognitive and language abilities do not process spoken utterances as “chunks” (Bavin et al., 2014, 2016; Brennan et al., 2019; Brock et al., 2008; Hahn et al., 2015; Pomper et al., 2021; Prescott et al., 2022; Venker et al., 2013, 2019; Zhou et al., 2019). To the contrary, these studies show that autistic children (like nonautistic children; Borovsky et al., 2012; Mani & Huettig, 2012) process spoken utterances incrementally (i.e., word-by-word) as they unfold. For example, when presented with an image of a bike and a cookie, autistic children, on average, began to look toward the bike when they heard the verb “ride” and to the cookie when they heard the verb “eat,” despite not yet hearing the complete utterances of “ride the bike” and “eat the cookie” (Prescott et al., 2022; Venker et al., 2019). These findings directly contradict the claim that autistic children process multiword utterances as “an unanalyzed whole” and may not understand or process individual words within longer utterances (Bavin et al., 2014, 2016; Brennan et al., 2019; Brock et al., 2008; Hahn et al., 2015; Pomper et al., 2021; Prescott et al., 2022; Venker et al., 2013, 2019; Zhou et al., 2019).

## Concern 2: The Conceptual Premise of GLP/NLA Is Fundamentally Flawed

GLP/NLA assumes that children’s production of delayed echolalia indicates a GLP style (Blanc et al., 2023). However, this assumption is flawed because spoken language production (i.e., expressive language) and language processing (an aspect of receptive language/comprehension) are separable constructs (ASHA, n.d.). Spoken language production is insufficient for determining what children understand or how they process language, yet the unique components and clinical strategies that comprise GLP/NLA rest on this flawed assumption. Although examining a child’s language production (e.g., through spontaneous language) is valuable for understanding spoken language development (Barokova & Tager-Flusberg, 2020; Plate, 2025), it is insufficient for assessing language processing and receptive language.

From a clinical perspective, it is natural to wonder: Why does it matter if an intervention approach has a fundamental conceptual flaw? What are the dangers of making claims about language processing based on spoken language production? The danger lies in the fact that this flawed assumption about children’s language processing is being used to make clinical recommendations related to assessment, goal writing, progress monitoring, and intervention strategies (Blanc et al., 2023). This may be a particularly dangerous assumption in the case of a nonspeaking child. If we were to use only what a nonspeaking child produces to determine how they process language, we are likely to severely underestimate their abilities. At present, GLP/NLA-specific recommendations—including labeling a child as a gestalt language processor, writing goals or tracking progress based on NLA stages, or using stage-based language modeling strategies—lack any supporting research evidence (see Concern 5). In general, clinicians should be wary of any approach that lacks a strong conceptual foundation, as such approaches are sometimes later found to be ineffective or even harmful (Finn et al., 2005; McCauley et al., 2009).

## Concern 3: GLP/NLA Assumes Autistic Children Do Not Understand Individual Words in Their Delayed Echoes, Which Presumes Incompetence and Is Inconsistent With Neurodiversity

Blanc et al. (2023) describe gestalts as “chunks” of language that children perceive as an “unanalyzed whole.” They propose that children “are not aware that a language gestalt could be made up of individual words that are combined” (Blanc et al., 2023, p. 1280; also see Peters, 1977). Not only is this assumption conceptually unjustified (see Concern 2) and refuted by existing research (see Concern 1), it is also inconsistent with neurodiversity (Hutchins et al., 2024; Venker & Lorang, 2024). Assuming that autistic children do not understand individual words in the delayed echoes they produce—without clear evidence that this is the case—is consistent with presuming incompetence. While not explicitly stated in the Blanc et al. article, GLP/NLA also appears to presume incompetence in other ways. For example, Blanc (an author on the viewpoint and the developer of GLP/NLA) claimed on a podcast that the “… cognitive capacity for grammar is born at Stage 3” (Prizant, 2024). In addition, in free professional development offerings, Blanc has also suggested that some academic concepts pertaining to literacy and math should not be introduced (or receive limited emphasis) until later NLA stages (Blanc, 2023, 2024b; Zachos, 2022). Withholding children’s exposure to specific types of language input, literacy or math experiences, or academic instruction could have serious consequences.

## Concern 4: The NLA Protocol’s Stage-Based Language Modeling Recommendations Are Unfounded and May Be Harmful

Despite being based on unsubstantiated claims and contradicted by existing research, the NLA protocol incorporates clinical recommendations for how adults should model language for children thought to be gestalt language processors (Blanc et al., 2023; also see Blanc, 2012). Depending on a child’s presumed stage, the NLA protocol makes unique language modeling recommendations, including that adults model language “without regard for grammar or word order” (Blanc et al., 2023, p. 1283), remove verbs from their spoken language, avoid single words, avoid questions, and not expand on children’s spoken utterances (Communication Development Center, 2024; Meaningful Speech, 2024b). However, no peer-reviewed, empirical research studies have investigated the effects of any of the language modeling strategies that are unique to the NLA protocol (Bryant et al., 2025). Furthermore, the strategies mentioned above are disconnected from and, in some cases, contradict well-established clinical practices (Bottema-Beutel & Kim, 2021; Crandall et al., 2019; Paul et al., 2018; Smith et al., 2023). In fact, research shows that parent verb use promotes language learning in autistic children (Crandall et al., 2019), that modeling some forms of ungrammatical language is associated with lower language abilities in autistic children (Venker et al., 2015), and that language expansions positively predict later language in autistic children (Smith et al., 2023). These unique language modeling strategies recommended by the NLA protocol are also based on flawed assumptions about how autistic children process and learn language (Hutchins et al., 2024; Venker & Lorang, 2024; see Concern 2) and may negatively affect children’s language development.

## Concern 5: GLP/NLA Is Not Supported by Peer-Reviewed, Empirical Research Evidence

In addition to being contradicted by existing research (see Concern 1), GLP and the NLA protocol lack supporting research evidence. A recent systematic review revealed that no published, peer-reviewed, empirical (i.e., data-based) studies have examined the effects of interventions based on GLP/NLA (Bryant et al., 2025). Similarly, no published, peer-reviewed, empirical studies have confirmed (or even examined) the proposal that GLP is a distinct style of language development common in autistic children or investigated the validity or reliability of clinical tools for identifying GLPs or the proposed NLA stages. Of course, a lack of published, peer-reviewed, empirical research support alone does not necessarily mean that a set of ideas is inaccurate or a set of intervention recommendations should not be used. However, SLPs should be highly skeptical when a lack of research support occurs alongside contradictory research evidence (see Concern 1), a flawed conceptual foundation (see Concern 2), and a clear disconnect from established practices (see Concern 4). Otherwise, we run the risk of prematurely integrating ideas into assessment and intervention that may have negative consequences for children and families (Bryant et al., 2025; Hutchins et al., 2024). Hutchins et al. (2024) have also expressed several similar critiques regarding GLP/NLA, such as the lack of well-defined concepts, the absence of external evidence supporting the claims surrounding GLP/NLA, and overall concerns about the theoretical basis of GLP/NLA. Moreover, research support is also an important consideration in the context of neurodiversity (Bottema-Beutel et al., 2023).

One potential point of confusion is that although we (and others; Beals, 2024; Bryant et al., 2025; Hutchins et al., 2024) conclude that GLP/NLA is not currently supported by research, some have asserted that it is. In their viewpoint, Blanc et al. (2023) state: “Following Prizant’s recommendation of a large-scale longitudinal study of the gestalt process, Blanc (2012) analyzed 15 years of clinical data collected from the language samples of dozens of autistic and neurotypical individuals who used a gestalt style of language development” (p. 1281). An addendum to Blanc’s (2012) book states: “As you may know, the NLA book was published in 2012, the result of 15 years of longitudinal clinical research in our clinic… “ (Blanc, 2024a). In addition, Blanc’s clinic website states: “Fifteen years of research backs up NLA” and “We hear stories every day about how well NLA supports are working all around the world, so we’re well beyond the need for ‘more research’ now… “ (Blanc, 2024d).

Given the stark difference between these characterizations and our own, it may be helpful to explain what led us to conclude that GLP/NLA lacks research support. In our view, it is justified to state that a set of ideas and clinical recommendations has research support when (a) relevant, empirical, peer-reviewed research studies are available and (b) the results of these studies directly support the claims being made. In our assessment, this is not the case for GLP/NLA because none of the sources commonly cited as research support for GLP/NLA meet these criteria. Most sources framed as providing research support for GLP/NLA are non–peer-reviewed books (Blanc, 2012; Peters, 1983) or opinion articles (Prizant, 1982, 1983). Although these sources are valuable, they do not constitute research support because they do not contain peer-reviewed, published, empirical research data. Peer review is an important component of research; without it, research studies are not held to any specific standards of credibility, ethics, or fidelity.

The sources that are empirical, peer-reviewed studies (Peters, 1977; Prizant & Rydell, 1984) are observational studies of delayed echolalia. Peters (1977) presented a case study of a child not identified as autistic who produced chunks of language that appeared to be unanalyzed wholes. Prizant and Rydell (1984) examined the production of delayed echolalia in three autistic children and identified the potential pragmatic functions of these delayed echoes. These studies are highly informative because they examine children’s production of delayed echolalia. However, the results of these studies do not support the specific claims and clinical recommendations that comprise GLP/NLA. Neither study provided evidence that GLP is a distinct style of language development, demonstrated how to identify children who may follow this hypothesized trajectory, identified NLA stages, tested whether NLA strategies are effective, or examined children’s language processing (see Concern 2). Thus, in our view, these studies—like other empirical studies of echolalia (Cohn et al., 2024; McAllister et al., 2025)—do not constitute direct research support for GLP/NLA.

While it is possible that studies are ongoing or in the peer review process, we currently have no peer-reviewed, published, empirical research studies that have examined or confirmed the set of ideas associated with GLP and the NLA protocol. In our view, when a new style of language development is being proposed and many of the recommended intervention strategies are contradictory to current practice, it is imperative that these ideas be supported by external research evidence.

## Conclusions

In this commentary, we described five concerns about GLP/NLA: that it is contradicted by existing research, has a fundamental conceptual flaw, presumes incompetence, recommends potentially harmful language modeling strategies, and lacks supporting research evidence. Given the severity of these concerns, we recommend that SLPs exercise caution when considering whether and how to implement NLA-specific strategies, such as labeling children as gestalt language processors, writing goals or tracking progress based on hypothesized NLA stages, or using stage-based NLA language modeling strategies. Avoiding unsupported and potentially harmful practices is one of the most powerful ways we, as SLPs, can apply our unique set of skills, knowledge, and clinical judgment toward providing evidence-based and neurodiversity-affirming practices for autistic children and their families.

At the same time, we hope that SLPs continue to use developmental strategies and neurodiversity-affirming practices such as those in Table 1. Although these strategies are associated with the NLA protocol, they can be used regardless of a clinician’s views about GLP/NLA. In fact, it is exciting to see the field moving toward honoring echolalia as a form of communication, alongside many other communication modalities. To avoid confusion, however, it may be beneficial to refer to practices and strategies in precise terms (e.g., “embracing echolalia,” “ listening to autistic voices,” and “following the child’s lead”), rather than describing them as GLP/NLA. Using precise terminology will help prevent miscommunications about the use of supported versus unsupported practices. Relatedly, we hope to see conversations shift from focusing on GLP/NLA as a whole to focusing on the individual ideas and clinical strategies that comprise it. Focusing on specific strategies and ideas may reveal a surprising amount of common ground among SLPs with seemingly opposing views (e.g., around the use of general developmental and neurodiversity-affirming strategies; see Table 1). It will also be beneficial to have thoughtful conversations about what practices and assumptions are truly neurodiversity affirming.

This is a critical point in our field. If we are to resolve the current controversy around GLP/NLA, we must engage in discussions that prioritize professionalism and respect. One way to move toward professional and respectful discussions is to take the time to thoughtfully consider points of view that differ from our own. It will be beneficial to avoid polarizing language; proponents of GLP/NLA, including Blanc, have previously referred to those with concerns about GLP/NLA as “naysayers” (Blanc, 2024c), which is unlikely to lead to respectful and productive conversations. It is also important to avoid invoking false dichotomies; for instance, it has been suggested that people who express concerns about GLP/NLA are proponents of applied behavior analysis, do not support neurodiversity, or do not listen to autistic voices.^2^ False dichotomies work in direct opposition to productive conversations.

We close with a reminder that the views in this commentary are our own—just as the views in Blanc et al.’s (2023) article are their own. Each reader must come to their own conclusion about GLP/NLA, and some will no doubt reach conclusions different from what we have reached. Perhaps the most powerful way to move forward is to remember that SLPs, regardless of how they view GLP/NLA, share a common goal: to provide individualized clinical services to support autistic people and their families in the most respectful, compassionate, and beneficial way.

## Figures and Tables

**Table 1.** General developmental clinical practices associated with (but not unique to) the Natural Language Acquisition (NLA) protocol.

General developmental practices
Establish trust and connection with children.
Collaborate with families.
Place connection at the forefront of communication.
Follow the child’s lead.
Acknowledge and honor all communication modes and attempts (including embracing echolalia).
Attempt to identify communicative functions of echolalia.
Incorporate language from home cultures.
Listen to autistic voices.

*Note*. We have no concerns about these clinical practices. It is our view that they can (and should) be used in interventions for autistic children, regardless of views about Gestalt Language Processing/NLA. All of these practices are considered general developmental practices because they are associated with many evidence-based interventions and are endorsed as neurodiversity-affirming practices (Binns et al., 2021; Donaldson et al., 2017; Gaddy & Crow, 2023; Ingersoll & Dvortcsak, 2019; Izuno-Garcia et al., 2023; Lai et al., 2020; Paul et al., 2018; Rogers et al., 2012; Schreibman et al., 2015; Sone et al., 2021; Sussman, 2012; Weitzman, 2013).

## Data Availability

No new data were analyzed for this commentary.

## Footnotes

1 The Communication Development Center is run by the first author of Blanc et al. (2023), and the associated website expands on many of the ideas in their publication. The original NLA protocol was published by Blanc (2012). Therefore, we include references to these materials in the current article in order to thoroughly describe the ideas associated with GLP/NLA.

2 We have also noticed a false dichotomy suggesting that calls for more research evidence are directly opposed to valuing lived experiences (Meaningful Speech, 2024a). Research evidence and lived experiences are both essential (Borneo, 2025).
